# In Vitro Corrosion and Cell Response of Hydroxyapatite Coated Mg Matrix in Situ Composites for Biodegradable Material Applications

**DOI:** 10.3390/ma12213474

**Published:** 2019-10-23

**Authors:** Nguyen Q. Cao, Hai M. Le, Khanh M. Pham, Nam V. Nguyen, Sachiko Hiromoto, Equo Kobayashi

**Affiliations:** 1 PHENIKAA Institute for Advanced Study, PHENIKAA University, Hanoi 100000, Vietnam; nguyen.caoquang@phenikaa-uni.edu.vn; 2 School of Materials Science and Engineering, Hanoi University of Science and Technology, Hanoi 100000, Vietnam; khanh.phammai@hust.edu.vn; 3 Institute of Traumatology and Orthopaedics, 108 Military Central Hospital, Hanoi 100000, Vietnam; namnguyenviet108@yahoo.de; 4 Corrosion Property Group, Research Center for Structural Materials, National Institute for Materials Science, Tsukuba 305-0047, Japan; hiromoto.sachiko@nims.go.jp; 5 Department of Materials Science and Engineering, Tokyo Institute of Technology, Tokyo 152-8550, Japan

**Keywords:** hydroxyapatite, Mg matrix composite, coating, corrosion resistance, cell viability

## Abstract

In this study, hydroxyapatite (HAp) coated Mg matrix composites were fabricated for biodegradable implant applications. Spark plasma sintering was employed to fabricate the Mg-10 wt% ZnO composite substrates. HAp was coated on the surface of the sintered composites and pure Mg by a chemical solution treatment. SEM and optical micrographs of coated samples showed that HAp grew homogeneously and formed a layer on the entire surface of both pure sintered Mg and Mg composites. The immersion and polarization test results demonstrated that the HAp coating significantly improved the corrosion resistance of the sintered composites. While the HAp coating layer is not effective in the improvement of the pure Mg substrate, cell culture test results revealed that the HAp coating improved cell adhesion and proliferation on the composites effectively through 72 h, while no cell could survive on the uncoated composites after 72 h. In addition, the corrosion tests and cell culture test results indicated that the composite with longer sintering time has better corrosion resistance and cell viability than those of the composite with shorter sintering time. The findings suggested that the HAp-coated Mg-10 wt% ZnO-2.5 h + 10 min composite is a high-potential candidate for biodegradable implant applications.

## 1. Introduction

Mg and Mg alloys are considered as high-potential materials for biodegradable implant applications [1,2,3]. Magnesium materials possess two major advantages for biodegradable implant applications: no need for secondary surgery after bone healing and avoiding stress shielding effect, for their equivalent Young’s modulus to bone [1,2,3,4]. In addition, Mg and Mg alloys are expected to be biocompatible because Mg is an essential element in the human body [5]. However, the main drawbacks of Mg and Mg alloys are their rapid corrosion rate, resulting in the deterioration of mechanical properties before bone healing and the formation of a gas cavity [6,7]. To solve this problem, the design and fabrication of new Mg matrix composites are a prospective approach. 

Dubey et al. fabricated Mg-3Zn-5HAp composite for biomedical applications [8]. The results suggested that the mechanical integrity of the fabricated Mg-3Zn-5HAp composite has been improved significantly compared to that of Mg-3Zn alloy after immersion tests for 3, 7, and 14 days in simulated body fluid. Witte et al. fabricated Mg matrix composite from AZ91 alloy and HAp as a biomaterial. The results demonstrated that HA effectively reinforced the mechanical properties and improved corrosion resistance of the fabricated composite compared to those of the AZ91 alloy [9]. Narita et al. fabricated Mg/β-TCP composites via the spark plasma sintering method for biodegradable material applications [10]. The findings indicated that the in vitro corrosion resistance and cytocompatibility of the fabricated Mg/β-TCP composites have been enhanced effectively compared to those of pure Mg. In our previous studies [11,12], Mg-ZnO in situ composites were fabricated successfully via spark plasma sintering (SPS) technique. With the reinforcement of in situ reaction products, including MgO, Zn, and intermetallic compounds, the fabricated composites showed superior mechanical properties to pure Mg and cast Mg alloys and improved the corrosion resistance for biodegradable material applications. In this study, aiming to further improve the corrosion resistance and cell viability of the fabricated Mg-ZnO in situ composites, surface coating was carried out on the composite substrates.

Surface coating is an effective method for improving the corrosion resistance of Mg materials [13,14,15,16,17,18]. The selection of an appropriate coating material is important because the coating material should be biocompatible. Among various coating materials, hydroxyapatite (HAp) is an effective coating material to improve both corrosion resistance and biocompatibility of Mg alloys [13,14,15,16,17,18]. Because HAp is the main component of human bone, it enhances the precipitation of calcium phosphate compounds on the surface from the simulated body fluids during biodegradation, which improved the corrosion resistance of Mg [19,20]. However, HAp coating on Mg matrix composites has not been investigated so far. In this study, HAp coating was conducted on the surface of the Mg matrix in situ composite substrates. The effect of the HAp coating layer on the corrosion behavior and the cell viability on the composites was examined. The uncoated samples were studied and reported in our previous work [12] and are not a part of this research.

## 2. Material and Methods 

### 2.1. Composite Synthesis

Mg powders with a purity of 99.5% and particle size of 180 *μ*m and ZnO powders with a purity of 99.9% and particle size of 1 *µ*m supplied by the KOJUNDO CHEMICAL LABORATORY CO., LTD—Japan were used for Mg matrix in situ composite syntheses. Mg-10 wt% ZnO was mixed homogeneously with zirconia balls (2.5 of ball to powder weight ratio) in an argon atmosphere using a planetary micro ball mill (Pulverisette 7, Fritsch, Idar, Oberstein, Germany) with a rotation speed of 500 rpm for 12 h. The mixed powders were set inside a tungsten–carbide die, 120 mm in height and 15 mm in inner diameter. The powder was compacted under 10 MPa for 1 min to form green compacts. The green compacts were then used for composite synthesis.

There were two types of composites fabricated. Mg-10ZnO-10 min was the composite sintered directly from the green compact by SPS (SPS-511S, Syntec Inc., Kanagawa, Japan) for 10 min, and Mg-10ZnO-2.5 h + 10 min was the composite sintered by two steps from the green compact. The first step was the sintering in a vacuum for 2.5 h without pressure [11,12]. The second step was the sintering by SPS for 10 min. In addition, pure Mg powder was also sintered by SPS for 10 min for comparison. The details of the fabrication process and sample names are shown in Table 1. All the sintering processes were performed in a vacuum furnace. After sintering processes, the samples were cooled to room temperature inside the vacuum furnace. 

### 2.2. HAp Coating

The sintered composites were machined into a disc shape with a size of *Φ*15 × *t*2 (mm). The machined samples were ground using SiC papers up to 4000 grit, ultrasonically cleaned with ethanol, and then dried in air. Treatment solution for HAp coatings was prepared from ethylenediaminetetraacetic acid calcium disodium salt hydrate (C_10_H_12_CaN_2_Na_2_O_8_, Ca-EDTA) solution with the concentration of 0.5 mol/L and potassium dihydrogen phosphate (KH_2_PO_4_) solution with the concentration of 0.5 mol/L, and sodium hydroxide (NaOH) solution was used for pH adjustment. The pH of the treatment solution was adjusted to 7.5. The discs were immersed in the treatment solution at 90 °C for 2 h for HAp coating. 

The coated samples were then characterized by X-ray diffractometry (XRD) (RINT2100, Rigaku, Tokyo, Japan) and scanning electron microscope (SEM) (Miniscope TM3000, Hitachi, Tokyo, Japan) to evaluate the growth of HAp coating layer.

### 2.3. Immersion Test

The HAp-coated samples were immersed in Hanks’ solution at 37 °C for 14 days to evaluate the corrosion properties. The composition of the Hanks’ solution is described in Table 2. The ratio of surface area to solution volume was 1 cm^2^: 50 mL. The immersion tests were conducted in triplicate for each sample condition. 

The amount of Mg^2+^ ions dissolved in the solution was quantified by a colorimetric method using Xylidyl blue-I [21,22]. In addition, hydrogen gas generated was collected by a burette with a funnel that was placed over the immersed sample. After 14 days of immersion, the immersed samples were retrieved from the solution for the surface characterization by SEM and energy dispersive X-ray spectrometer (EDS) (Quantax 70, Bruker, Billerica, MA, USA). 

### 2.4. Polarization Tests 

Polarization tests were carried out in Hanks’ solution. The surface was coated with epoxy resin to expose the measurement area of 1 cm^2^ to Hanks’ solution at 37 °C. The open circuit potential was measured for 1500 s. Subsequently, potentiodynamic polarization curves were measured in the potential range from −0.1 V vs. open circuit potential (*E*_ocp_) at a scan rate of 1 mV/s. A reference and counter electrode were saturated Ag/AgCl and Pt electrodes, respectively. 

### 2.5. Cell Viability Test

The HAp-coated and uncoated samples were then used for the evaluation of cell viability by cell culture tests. The cell culture tests were carried out using mouse osteoblastic cell line MC3T3-E1 supplied by RIKEN Cell Bank (Cell No. RBRC-RCB1126) in α medium supplemented with 10 vol.% fetal bovine serum (α-MEM + FBS) at 37 ± 0.5 °C under a 5% CO_2_ atmosphere for 24, 48, and 72 h, respectively. The initial cell density in cell culture tests was 25,000 cells/cm^2^.

After incubation for 24, 48, and 72 h, the samples were retrieved from the medium, and the cells were stained with Giemsa for observation of cell proliferation and morphology. In this study, the cell viability was examined qualitatively; therefore, the Giemsa stain method was used for its low cost and simplicity. 

## 3. Result and Discussion

### 3.1. Composition and Microstructure of Sintered Composites

**Figure 1:**
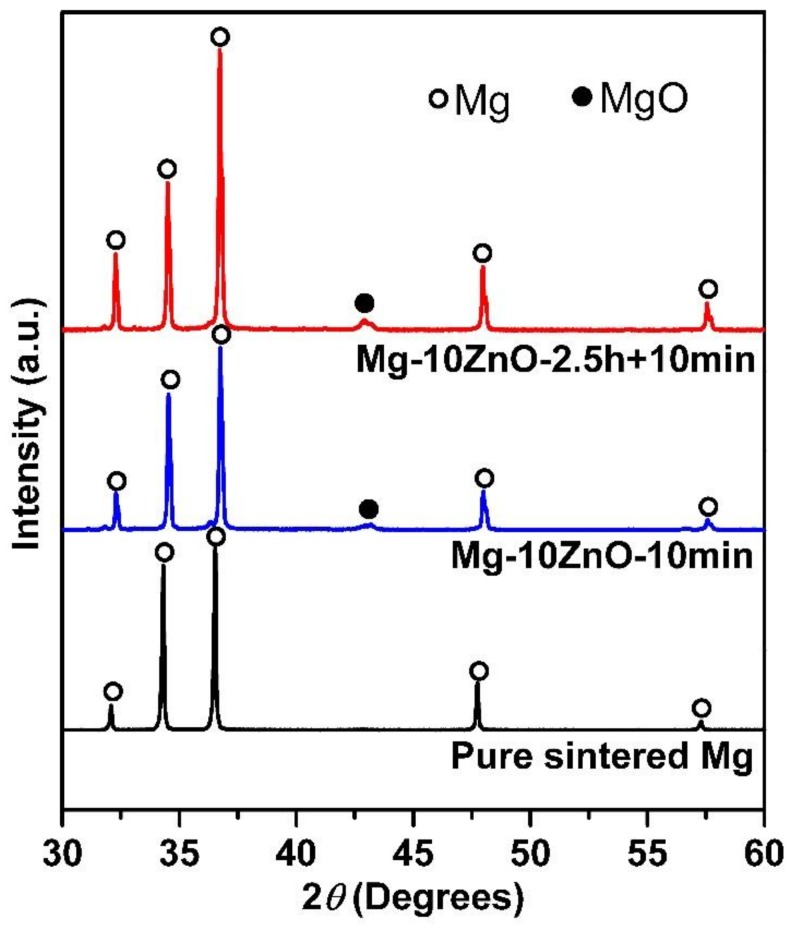
XRD patterns of sintered samples.

XRD patterns of the sintered Mg, Mg-10ZnO-10 min, and Mg-10ZnO-2.5 h + 10 min samples are shown in Figure 1. There are only peaks of Mg on the XRD pattern of sintered pure Mg, while peaks of MgO appeared additionally to the peaks of Mg on the XRD patterns of the composites. The authors [11,12] demonstrated that the following in situ reactions occurred during sintering mixed Mg and ZnO powders from 450 °C to 550 °C.

(1) $${\mathrm{Mg}}_{\left(s\right)}\;+\;{\mathrm{ZnO}}_{\left(s\right)}\overset{450-500\;℃}{\to }{\mathrm{MgO}}_{\left(s\right)}\;+\;{\mathrm{Zn}}_{(l),}$$

(2) $${\mathrm{Mg}}_{\left(s\right)}\;+\;{\mathrm{Zn}}_{\left(l\right)}\overset{500-550\;℃}{\to }\mathrm{Mg}-{\mathrm{Zn}}_{\left(l\right)},$$

(3) $$\mathrm{Mg}-{\mathrm{Zn}}_{\left(l\right)}\overset{\mathrm{Cooling}}{\to }{\mathrm{Mg}}_{\left(s\right)}\;+\;{\mathrm{Mg}}_{x}{\mathrm{Zn}}_{y},$$

where the subscripts (s) and (l) refer to solid state and liquid state, respectively, and Mg_x_Zn_y_ corresponds to MgZn, Mg_2_Zn_3_, and Mg_7_Zn_3_ intermetallic compounds.

The in situ reaction (1) explains the existence of MgO as an in situ reaction product. Therefore, the peaks of MgO appeared on the XRD patterns of the sintered composites. Based on the Mg-Zn binary phase diagram [23], the reactions (2) and (3) occurred reasonably. Therefore, Zn and Mg-Zn intermetallic compounds were formed as a part of in situ reaction products in the sintered composites [11,12]. The amount of newly formed MgO, Zn, and intermetallic compounds in the Mg-10ZnO-10 min and Mg-10ZnO-2.5 h + 10 min composites are different due to different sintering time [12].

**Figure 2:**
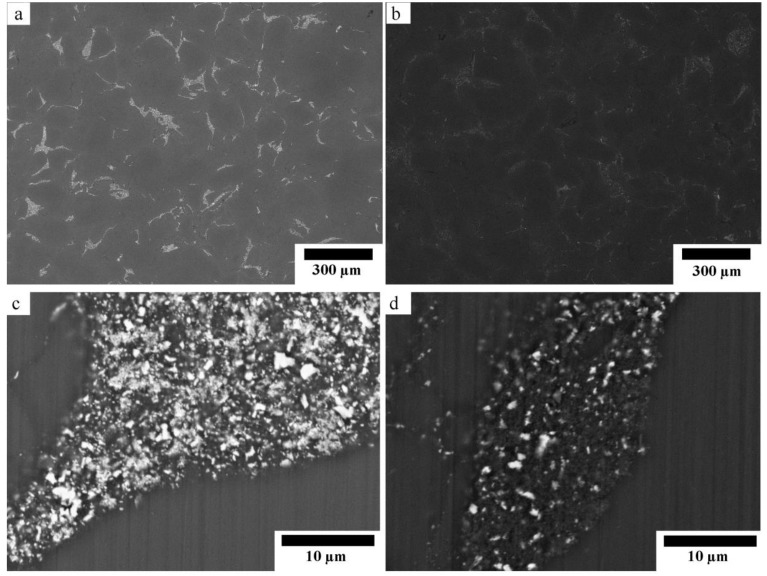
SEM micrographs of the sintered composites: (**a**,**c**) Mg-10ZnO-10 min, (**b**,**d**) Mg-10ZnO-2.5 h + 10 min.

Figure 2 shows the SEM micrographs of the sintered composites with different magnifications. Figure 2a,b show that both composites have two identical phases of Mg matrix and dispersion of reinforcement. The reinforcement phase contained randomly dispersed white particles which were remained ZnO [12]. The amount of remained ZnO in the Mg-10ZnO-10 min sample was much higher than in the Mg-10ZnO-2.5 h + 10 min sample, as shown in Figure 2c,d because the sintering time of the former was only 10 min which was remarkably shorter than the total sintering time of 160 min of the latter. The in situ reactions (1) and (2) progressed further with an increase in sintering time. Therefore, the amount of remained ZnO in the single-step composite became larger than that in the double-step composites. The smaller amount of remained ZnO corresponded to the progress of the reactions (1) and (2). This fact indicates that the amount of produced MgO and intermetallic compounds in the double-step composites was larger than those in the single-step sintering composite [12].

### 3.2. HAp Growth on Surface of Sintered Samples

**Figure 3:**
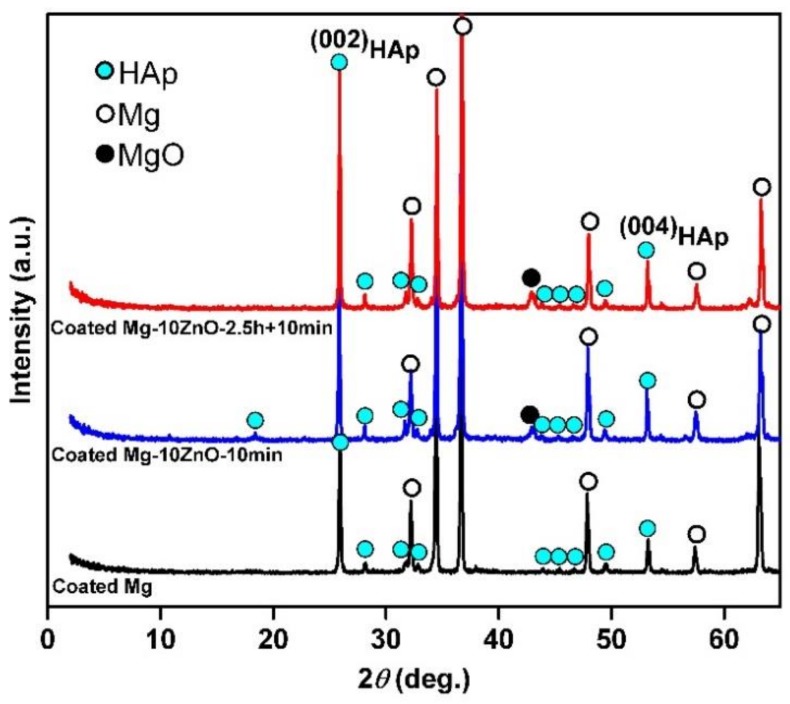
XRD patterns of hydroxyapatite (HAp)-coated samples.

Figure 3 shows XRD patterns of HAp-coated Mg, Mg-10ZnO-10 min, and Mg-10ZnO-2.5 h + 10 min samples. All samples show high intensity peaks of HAp. This demonstrated that HAp grew successfully on the surface of all samples. On all substrates, the (002)_HAp_ peak showed a higher intensity than the other HAp peaks. This suggested that the (002) plane of HAp grew preferentially. This preferential orientation of the (002) plane of HAp to the surface is similar to that of HAp on Mg alloys in previous studies [13,17].

**Figure 4:**
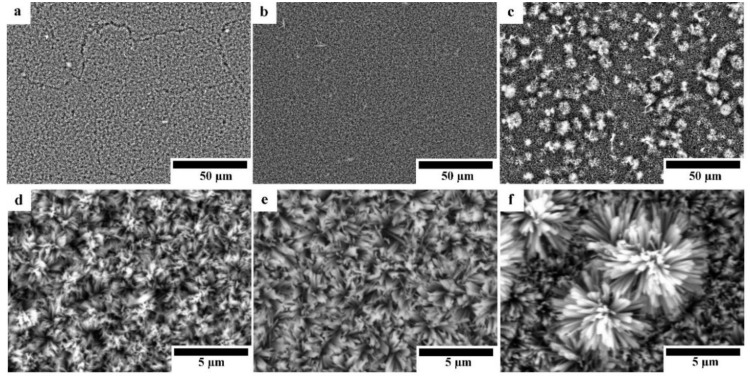
SEM micrographs of HAp-coated samples: (**a**,**d**) HAp-coated Mg, (**b**,**e**) HAp-coated Mg-10ZnO-10 min, (**c**,**f**) HAp-coated Mg-10ZnO-2.5 h + 10 min.

Figure 4 shows the SEM micrographs of HAp-coated Mg, Mg-10ZnO-10 min, and Mg-10ZnO-2.5 h + 10 min samples with different magnifications. All surfaces were covered with HAp layers with an outside porous structure consisting of rod-like crystals. Because the morphology of the outside surface of the HAp coatings in this study was similar to that formed on pure Mg and AZ31 [13,17], the HAp layers in this study necessarily had a continuous inner layer, which shows corrosion protection ability. On pure Mg, cracking of the HAp layer was observed probably along the grain boundaries in Figure 4a. On Mg-10ZnO-2.5 h + 10 min, flower-like agglomerates randomly appeared on the top surface. The flower-like HAp agglomerates presumably nucleated from MgO and MgZn intermetallic compounds rich regions on this sample. 

**Figure 5:**
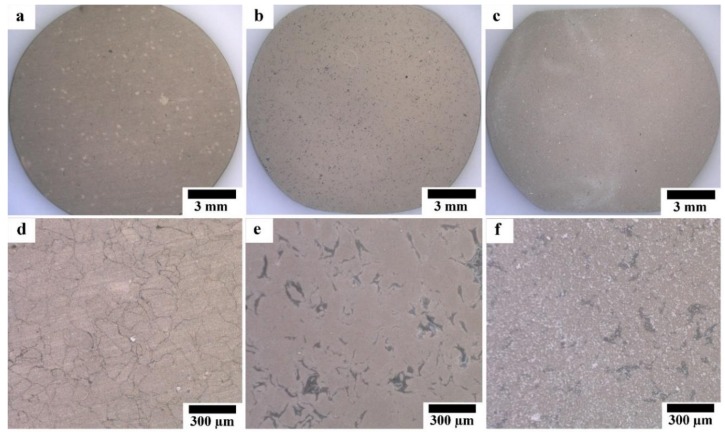
Optical micrographs of HAp-coated samples: (**a**,**d**) HAp-coated Mg, (**b**,**e**) HAp-coated Mg-10ZnO-10 min, (**c**,**f**) HAp-coated Mg-10ZnO-2.5 h + 10 min.

Optical micrographs of coated samples are shown in Figure 5. As shown in Figure 5a,d, the HAp layer grows homogeneously on the surface of Mg substrate; while the grain boundaries between Mg particles are observed through the HAp layer, indicating that HAp layer on the Mg substrate was transparent and the grain boundaries were preferentially corroded in the HAp coating solution. No grain boundary was observed through the HAp layer on the surface of HAp-coated composites; however, the grey grains of the second phase are observed through the transparent HAp layer. This appearance indicates that the second phase was preferentially corroded in the HAp coating solution due to the existence of MgO and intermetallic compounds. The macroscopically uniform coating shown in Figure 5a,b indicates that the apparent micro galvanic corrosion in the HAp coating solution did not influence the macroscopic morphological uniformity of the HAp layer on Mg and Mg-10ZnO-10 min samples. The HAp-coated Mg-10ZnO-2.5 h + 10 min shows the uniform coating layer with randomly distributed white agglomerates. These white agglomerates corresponded to flower-like crystals on the SEM images in Figure 4c,f. The grains of the second phase were preferentially corroded on Mg-10ZnO-2.5 h + 10 min as well as those on Mg-10ZnO-10 min; however, the distribution of white agglomerates was macroscopically inconsistent to that of the corroded second phase. Although the formation of HAp crystal agglomerates was observed, the composite surface was completely covered with the HAp layer.

### 3.3. Static Corrosion Behavior in Hanks’ Solution

**Figure 6:**
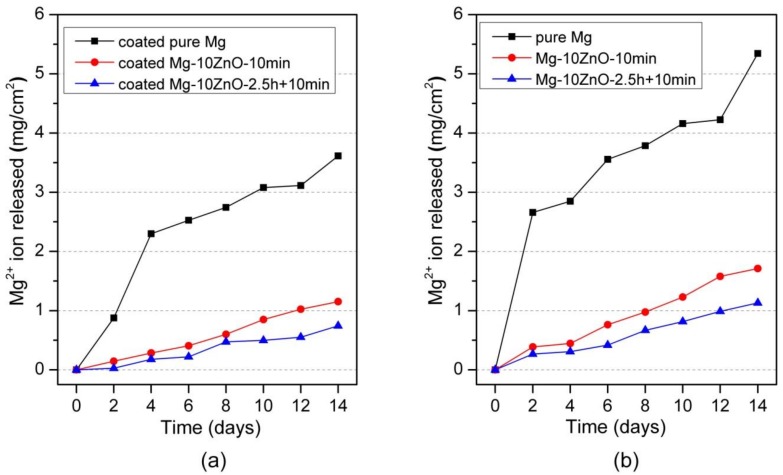
Mg^2+^ ion release in Hanks’ solution during immersion test, (**a**) the result of HAp coated samples in this study, and (**b**) the result of uncoated samples was cited from [12].

Figure 6a shows the amount of Mg^2+^ ion released per cm^2^ as a function of the immersion period of HAp-coated in Hanks’ solution. The coated Mg-10ZnO-2.5 h + 10 min samples showed the lowest corrosion rate, while the coated Mg exhibited the highest amount of Mg^2+^ ion released within the coated samples. This indicates that the corrosion resistance of HAp-coated composites was dominated by the corrosion resistance of the substrate composite because Mg-10ZnO-2.5 h + 10 min substrate had the highest corrosion resistance as shown in Figure 6b referred from [12].

**Figure 7:**
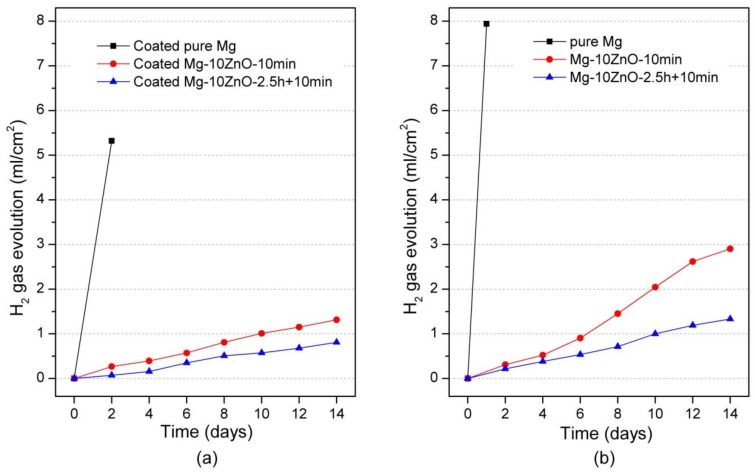
Hydrogen gas evolution during the immersion test, (**a**) the result of HAp coated samples in this study, and (**b**) the result of uncoated samples was cited from [12].

Figure 7a shows the hydrogen-evolution behavior of HAp-coated samples during 14 days-immersion in Hanks’ solution. The hydrogen-evolution behavior showed the same order of corrosion resistance between samples to the Mg^2+^ ion release behavior. Specifically, the coated Mg-10ZnO-2.5 h + 10 min samples showed the smallest amount of hydrogen gas release while the coated Mg exhibited the largest amount of hydrogen gas release. This result confirms that the HAp-coated Mg-10ZnO-2.5 h + 10 min samples showed the lowest corrosion rate, while the HAp-coated pure Mg showed the highest corrosion rate.

**Figure 8:**
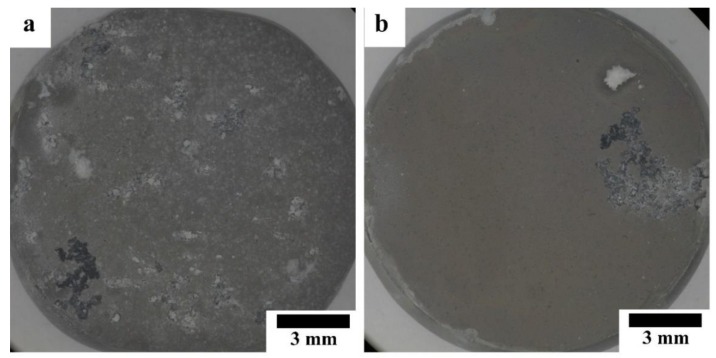
Morphology of HAp-coated composites after 14 days of immersion: (**a**) immersed HAp-coated Mg-10ZnO-10 min, (**b**) immersed HAp-coated Mg-10ZnO-2.5 h + 10 min.

Figure 8 shows optical micrographs of the HAp-coated Mg-10ZnO-10 min and Mg-10ZnO-2.5 h + 10 min samples immersed in Hanks’ solution for 14 days. The coated pure Mg samples were fractured during the immersion test due to the poor corrosion resistance of this sample. The coated Mg-10ZnO-10 min showed many severe localized corrosion sites and a significant amount of corrosion product deposited, while the coated Mg-10ZnO-2.5 h + 10 min sample showed a few localized corrosion sites. This result indicates that the coated Mg-10ZnO-10 min had many corrosion initiation sites like defects of the coating and/or the inhomogeneous sites of the substrate composite. The corrosion progress led to the increase in corrosion product, which caused the breakage and delamination of the HAp coating. A smaller number of corrosion sites of the coated Mg-10ZnO-2.5 h + 10 min samples indicates that the corrosion initiation sites decreased by the long time sintering. 

**Figure 9:**
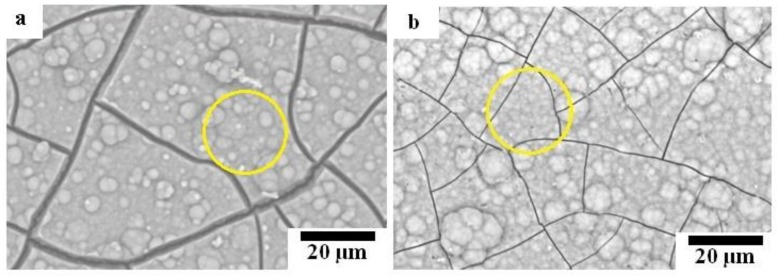
SEM micrographs of HAp-coated composites after 14 days of immersion: (**a**) immersed HAp-coated Mg-10ZnO-10 min, (**b**) immersed HAp-coated Mg-10ZnO-2.5 h + 10 min.

Figure 9 shows SEM images of the non-corrosion regions of HAp-coated composites immersed in Hanks’ solution. The surface of both samples was covered by corrosion product. The EDS analysis of the areas marked with yellow circles in Figure 9 is shown in Table 3. From EDS analysis results, calcium phosphate compounds were the main corrosion product. In the case of immersed HAp-coated Mg-10ZnO-2.5 h + 10 min sample, HAp was the main constituent of the deposited calcium phosphate layer since the atomic percent ratio of Ca and P (Ca/P ratio) was approximately 1.72 that is similar to 1.67 of HAp [19]. As for the HAp-coated Mg-10ZnO-10 min sample, amorphous calcium phosphate was presumably the main component of deposited calcium phosphate because the Ca/P ratio was ca. 1.40.

**Figure 10:**
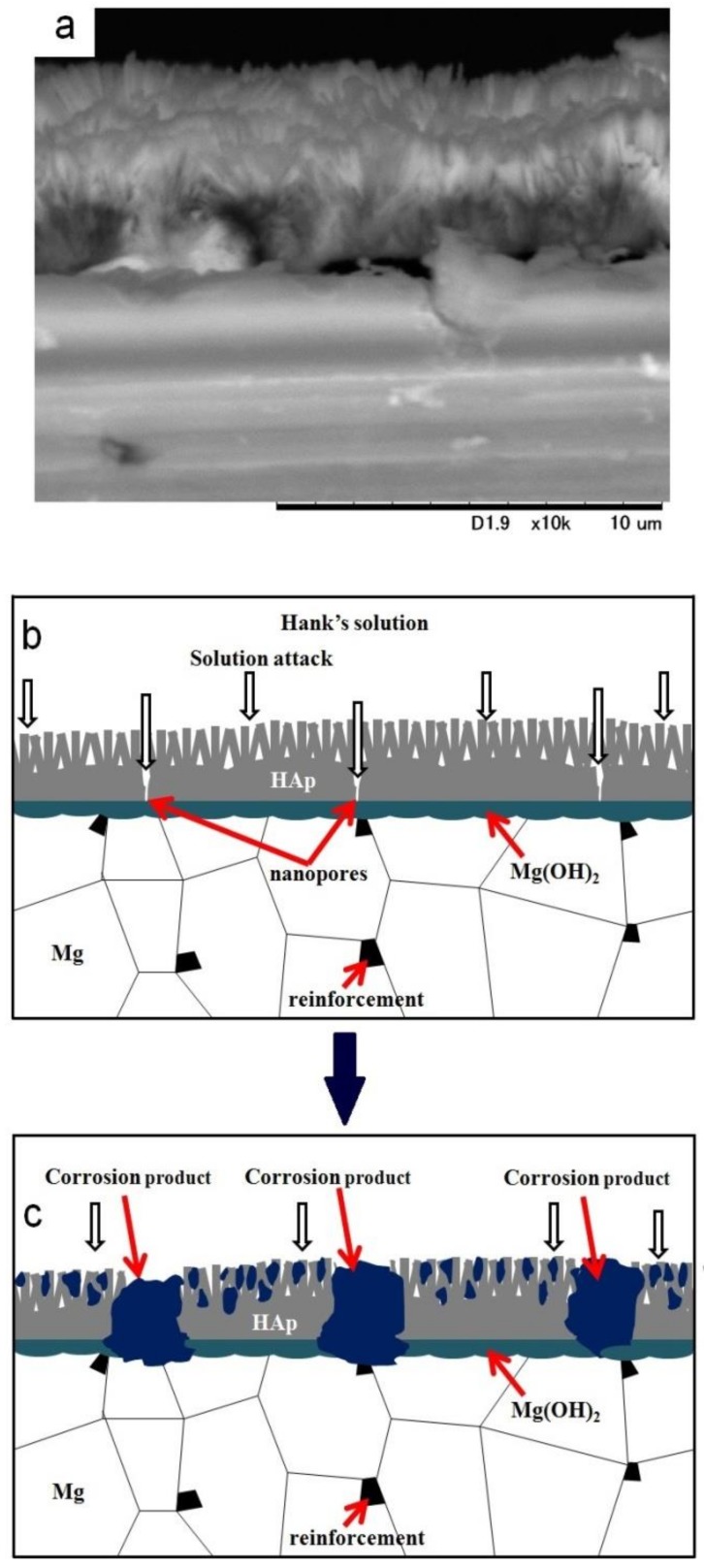
SEM image of the cross-section of a HAp-coated Mg-10ZnO-2.5 h + 10 min sample (**a**) and illustration of the corrosion process of the HAp-coated composites (**b**) and (**c**).

It has been reported that HAp coating improved the corrosion resistance of materials effectively as a permeation barrier against solution [13,17,18]. However, it has been reported that nanopores always existed in the HAp coating layer [17,18], and HAp is a hydrophilic compound. Since the HAp layer on sintered pure Mg showed cracking along grain boundaries, defects of the HAp layer could be formed due to grain boundaries, the second phase, and the drying procedure after the coating. Therefore, the solution permeates through the HAp coating preferentially via the defects and the corrosion of the substrate initiates. Figure 10 shows an SEM image of the cross-section of the HAp-coated Mg-10ZnO-2.5 h + 10 min sample and describes the corrosion process of the HAp-coated composites in Hanks’ solution. The existence of Mg(OH)_2_ as an intermediate layer was demonstrated by Tomozawa et al. [17]. As can be seen, corrosion starts on the substrate via defects of the HAp coating. The corrosion of the substrate produced corrosion product at the interface of the HAp layer and the substrate. The micro galvanic corrosion due to the second phase, and grain boundaries could promote the corrosion of the substrate. The corrosion progress caused the increase in surface pH, leading to the deposition of calcium phosphate compounds over the HAp layer. Concurrently, the corrosion progress at the interface increased the amount of corrosion product and gradually broke the dense inner layer of HAp, leading to the macroscopic localized corrosion. When the HAp layer was broken, the bare metal surface was exposed to the solution, and rapid corrosion produced a large amount of corrosion product at the corrosion sites. The formation of corrosion product filling the corrosion pits and the remaining dense HAp layer can effectively prevent severe corrosion occurring afterward.

### 3.4. Polarization Tests

**Figure 11:**
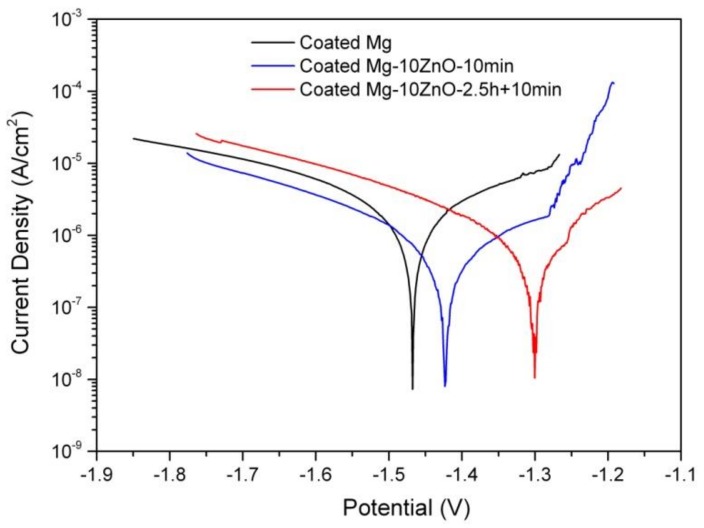
Potentiodynamic polarization curves of as-fabricated samples in Hanks’ solution.

Figure 11 shows the polarization curves of the HAp-coated Mg, Mg-10ZnO-10 min, and Mg-10ZnO-2.5 h + 10 min samples in Hanks’ solution. The coated Mg-10ZnO-2.5 h + 10 min sample exhibited the lowest corrosion current density (*I*_corr_) and the highest corrosion potential (*E*_corr_), while the coated Mg specimen showed the highest *I*_corr_ and the lowest *E*_corr_. These results agreed with the results of the immersion test: the coated Mg-10ZnO-2.5 h + 10 min exhibited the highest corrosion resistance, while the coated Mg showed the highest corrosion rate. 

### 3.5. Cell Viability

**Figure 12:**
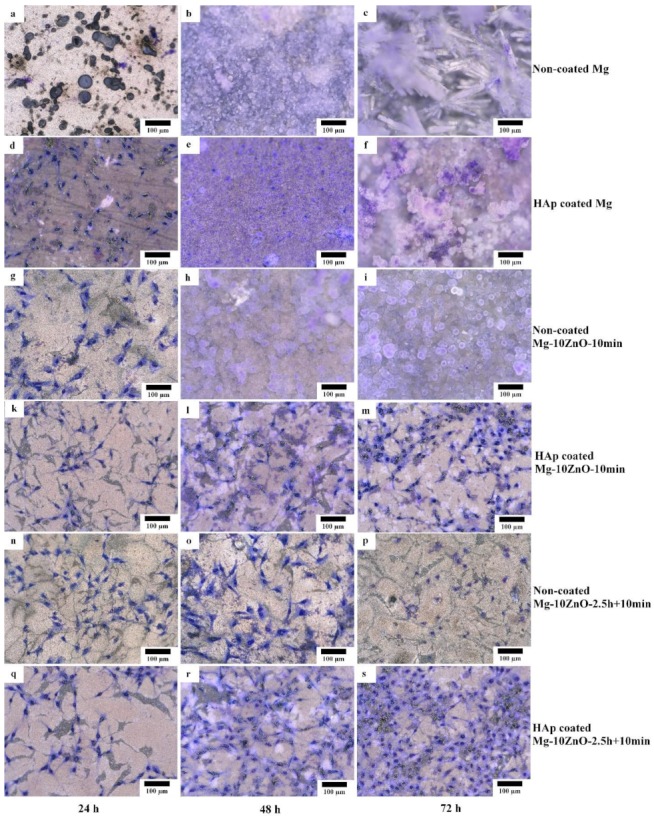
Morphology of the coated and uncoated samples after cell culture test for 24, 48, and 72 h, respectively. (**a,b,c**) uncoated Mg, (**d,e,f**) HAp coated Mg, (**g,h,i**) uncoated Mg-10ZnO-10min, (**k,l,m**) HAp coated Mg-10ZnO-10min, (**n,o,p**) uncoated Mg-10ZnO-2.5h+10min, (**q,r,s**) HAp coated Mg-10ZnO-2.5h+10min.

Figure 12 shows the optical micrographs of the HAp-coated and uncoated samples after cell culture for 24, 48, and 72 h. Between the samples with the same sintering condition and composition, the cell viability on the HAp-coated samples was much higher than that on uncoated samples. As for the sintered pure Mg, no cell survived on the uncoated sample after only 24 h. Many cells survived on the HAp-coated Mg sample after 24 h of culture even though some localized corrosion occurred. However, after 48 h, corrosion occurred over almost the entire surface of the HAp-coated Mg sample, and corrosion product covered the whole surface. No cell remained on this sample. After 72 h, the HAp-coated Mg sample was corroded more severely, and a thick layer of corrosion product covered the entire surface of this sample.

On the uncoated Mg-10ZnO-10 min and Mg-10ZnO-2.5 h + 10 min composites, a part of the cells survived for 24 and 48 h, respectively. After 48 h, on the uncoated Mg-10ZnO-10 min, corrosion product almost covered the surface, and no cells remained. After 72 h on the uncoated Mg-10ZnO-2.5 h + 10 min, significant corrosion product was not observed, while the number of cells decreased. Both HAp-coated Mg-10ZnO-10 min and Mg-10ZnO-2.5 h + 10 min samples showed very good cell viability. No obvious corrosion was observed on both HAp-coated composites after 72 h. These facts indicate that HAp coating is very effective in improving the corrosion resistance of the as-fabricated composites, resulting in the significant improvement of cell viability on the composites. The cell density on both composites increased gradually with an increase in cell culture time, suggesting the good biocompatibility of the HAp-coated surfaces. As for the comparison between two composites, cell density on the HAp-coated Mg-10ZnO-2.5 h + 10 min sample was higher than that on the HAp-coated Mg-10ZnO-10 min sample. 

As demonstrated in previous studies, HAp improves the biocompatibility and bioactivity of the materials [20,24,25]. The formation of HAp coating is not only for reducing the corrosion rate of the substrate but also for promoting new bone growth [20,25,26]. In this study, Figure 12 shows that osteoblast cells can grow well on the whole surface of the HAp-coated composites.

**Figure 13:**
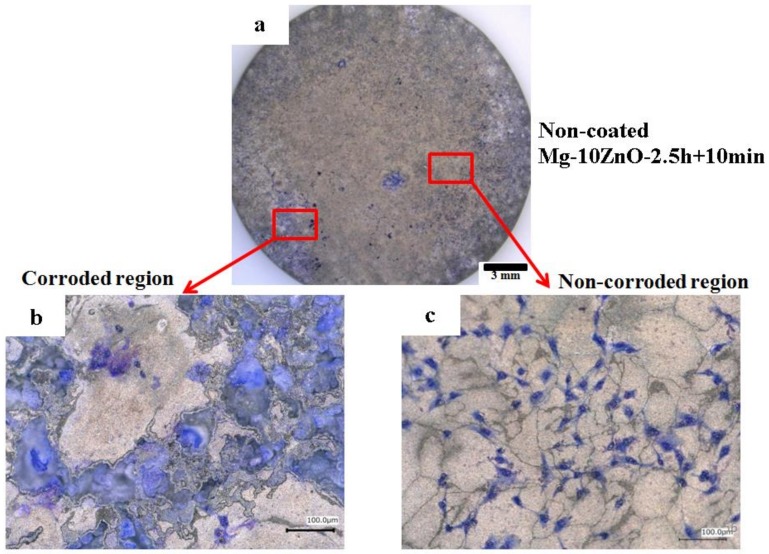
Morphology of the uncoated Mg-10ZnO-2.5 h + 10 min sample after 24 h of cell culture, (**a**) full surface image, (**b**) corroded region, (**c**) non-corroded region.

**Figure 14:**
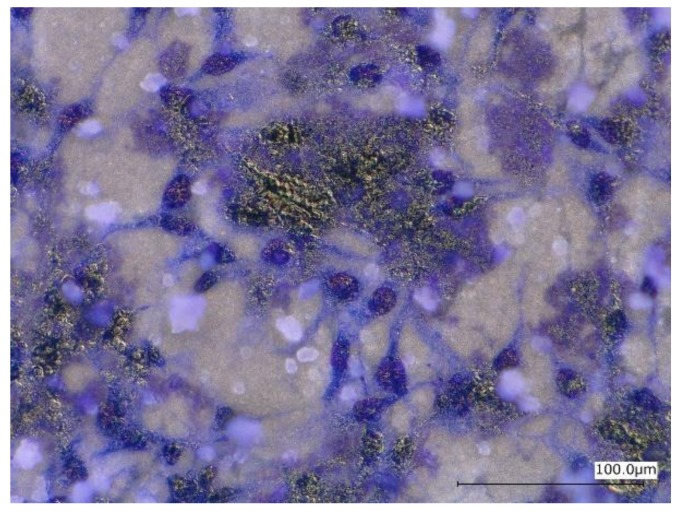
Morphology of the HAp-coated Mg-10ZnO-2.5 h + 10 min sample after 72 h of cell culture.

Figure 13 shows optical micrographs of the uncoated Mg-10ZnO-2.5 h + 10 min sample after 24 h of cell culture. Some local corrosion occurred on the surface of the uncoated sample. High magnification images show that almost no cell survived in the corroded region (Figure 13b), and cells extended well on the non-corroded region (Figure 13c). This result is consistent with the previous studies, which revealed that the corrosion rate of Mg alloys strongly influenced the cell viability [6,25,26,27,28]. When the sample was immersed in the cell culture medium, local corrosion occurred immediately, and hydrogen gas evolution with a high rate was observed. The hydrogen gas evolution at a high rate prevented a part of cells from adhering to the surface of the sample [6,27,28]. Song postulated that the hydrogen gas evolution rate of 0.01 mL/cm^2^/day is a tolerated rate for cell proliferation [27]. In addition, a part of cells once adhered to the surface but detached due to the occurrence of corrosion around the cells. In this work, the HAp coating layer effectively prevented the hydrogen gas evolution at the initial time of cell culture. Therefore, cells adhered well to the surface of the HAp-coated sample. Figure 14 shows that corrosion product deposited on the surface of HAp-coated Mg-10ZnO-2.5 h + 10 min after 72 h of cell culture. However, cells can proliferate over and around the corrosion product. The HAp coating retarded the corrosion (pH increase and hydrogen gas evolution) at a certain tolerable level for cells, allowing cells to proliferate until 72 h even on the corrosion product. These results demonstrated that the retardation of corrosion is very important for cell viability, and HAp coating is an effective method for improving cell viability on Mg matrix composites.

## 4. Conclusions

HAp coating layer was formed successfully on the sintered Mg-ZnO in situ composites with different sintering conditions by a chemical solution treatment. The HAp layer effectively improved the corrosion resistance and the cell viability on the surface of the composites regardless of the sintering condition. The HAp-coated Mg-ZnO composite with longer total sintering time showed higher corrosion resistance and cell viability than those with shorter sintering time. The HAp coating layer effectively retarded the corrosion enough to improve the cell viability on the surface of the composites, although the corrosion resistance of HAp-coated composite depended on the corrosion resistance of uncoated composites. The results suggested that HAp-coated Mg matrix in situ composites are promising materials for biodegradable implant applications.

## Figures and Tables

**Table 1:** Name, composition, and synthesis conditions of the samples.

Sample Name	Composition	Sintering in a Vacuum (T = 550 °C, P = 0 MPa)	Sintering by SPS (T = 550 °C, P = 50 MPa)
Mg	Pure Mg powder	0	10 min
Mg-10ZnO-10 min	Mg-10 mass% ZnO	0	10 min
Mg-10ZnO-2.5 h + 10 min	Mg-10 mass% ZnO	2.5 h	10 min

**Table 2:** Composition of Hanks’ solution used for immersion tests.

Reagent	NaCl	KCl	Na_2_HPO_4_.H_2_O	KH_2_PO_4_	MgSO_4_.7H_2_O	NaHCO_3_	CaCl_2_
Concentration (g/L)	8	0.4	0.06	0.06	0.2	0.35	0.14

**Table 3:** EDS analysis results on the surfaces of immersed HAp-coated composites.

Element	Immersed HAp-Coated Mg-10ZnO-10 min	Immersed HAp-Coated Mg-10ZnO-2.5 h + 10 min
Oxygen (at %)	66.0	67.0
Calcium (at %)	17.5	19.0
Phosphorus (at %)	12.5	11.0
Magnesium (at %)	4.0	3.0
Ca/P ratio	1.40	1.72

## References

[1] Wu G., Ibrahim J.M., Chu P.K. (2013). Surface design of biodegradable magnesium alloys—A review. Surf. Coat. Technol..

[2] Li L.Y., Cui L.Y., Zeng R.C., Li S.Q., Chen X.B., Zheng Y., Kannan M.B. (2018). Advances in functionalized polymer coatings on biodegradable magnesium alloys—A review. Acta Biomater..

[3] Agarwal S., Curtin J., Duffy B., Jaiswal S. (2016). Biodegradable magnesium alloys for orthopaedic applications: A review on corrosion, biocompatibility and surface modifications. Mater. Sci. Eng. C.

[4] Staiger M.P., Pietak A.M., Huadmai J., Dias G. (2006). Magnesium and its alloys as orthopedic biomaterials: A review. Biomaterials.

[5] Jahnen-Dechent W., Ketteler M. (2012). Magnesium basics. CKJ Clin. Kidney J..

[6] Witte F., Kaese V., Haferkamp H., Switzer E., Meyer-Lindenberg A., Wirth C.J., Windhagen H. (2005). In vivo corrosion of four magnesium alloys and the associated bone response. Biomaterials.

[7] Kirkland N.T., Lespagnol J., Birbilis N., Staiger M.P. (2010). A survey of bio-corrosion rates of magnesium alloys. Corros. Sci..

[8] Dubey A., Jaiswal S., Lahiri D. (2019). Mechanical Integrity of Biodegradable Mg–HA Composite During In Vitro Exposure. J. Mater. Eng. Perform..

[9] Witte F., Feyerabend F., Maier P., Fischer J., Sto¨rmer M., Blawert C., Dietzel W., Hortb N. (2007). Biodegradable magnesium–hydroxyapatite metal matrix composites. Biomaterials.

[10] Narita K., Tian Q., Johnson I., Zhang C., Kobayashi E., Liu H. (2019). Degradation behaviors and cytocompatibility of Mg/β-tricalcium phosphate composites produced by spark plasma sintering. J. Biomed. Mater. Res. Part B Appl. Biomater..

[11] Cao N.Q., Narita K., Kobayashi E., Sato T. (2016). Evolution of the microstructure and mechanical properties of Mg-matrix in situ composites during spark plasma sintering. Powder Metall..

[12] Cao N.Q., Pham D.N., Kai N., Dinh H.V., Hiromoto S., Kobayashi E. (2017). In Vitro Corrosion Properties of Mg Matrix In Situ Composites Fabricated by Spark Plasma Sintering. Metals.

[13] Hiromoto S., Tomozawa M. (2011). Hydroxyapatite coating of AZ31 magnesium alloy by a solution treatment and its corrosion behavior in NaCl solution. Surf. Coatings Technol..

[14] Tang H., Xin T.Z., Luo Y., Wang F.P. (2013). In vitro degradation of AZ31 magnesium alloy coated with hydroxyapatite by sol–gel method. Mater. Sci. Technol..

[15] Kaabi Falahieh Asl S., Nemeth S., Tan M.J. (2015). Improved corrosion protection of magnesium by hydrothermally deposited biodegradable calcium phosphate coating. Mater. Chem. Phys..

[16] Kim S.Y., Kim Y.K., Ryu M.H., Bae T.S., Lee M.H. (2017). Corrosion resistance and bioactivity enhancement of MAO coated Mg alloy depending on the time of hydrothermal treatment in Ca-EDTA solution. Sci. Rep..

[17] Tomozawa M., Hiromoto S. (2011). Growth mechanism of hydroxyapatite-coatings formed on pure magnesium and corrosion behavior of the coated magnesium. Appl. Surf. Sci..

[18] Yang H., Xia K., Wang T., Niu J., Song Y., Xiong Z., Zheng K., Wei S., Lu W. (2016). Growth, in vitro biodegradation and cytocompatibility properties of nano-hydroxyapatite coatings on biodegradable magnesium alloys. J. Alloys Compd..

[19] Kuwahara H., Al-Abdullat Y., Mazaki N., Tsutsumi S., Aizawa T. (2001). Precipitation of Magnesium Apatite on Pure Magnesium Surface during Immersing in Hank’s Solution. Mater Trans..

[20] Hiromoto S., Shishido T., Yamamoto A., Maruyama N., Somekawa H., Mukai T. (2008). Precipitation control of calcium phosphate on pure magnesium by anodization. Corros. Sci..

[21] Mann C.K., Yoe J.H. (1956). Spectrophotometric Determination of Magnesium with Sodium 1-Azo-2-hydroxy-3-(2,4-dimethylcarboxanilido)-naphthalene-1´-(2-hydroxybenzene-5-sulfonate). Anal. Chem..

[22] Watanabe H., Tanaka H. (1977). Dual-wavelength spectrophotometric determination of magnesium with xylidyl blue I and nonionic surfactant. Bunseki Kagaku..

[23] Baker H. (1992). ASM Handbook.

[24] Husak Y., Solodovnyk O., Yanovska A., Kozik Y., Liubchak I., Ivchenko V., Mishchenko O., Zinchenko Y., Kuznetsov V., Pogorielov M. (2018). Degradation and In Vivo Response of Hydroxyapatite-Coated Mg Alloy. Coatings.

[25] Hiromoto S., Inoue M., Taguchi T., Yamane M., Ohtsu N. (2015). In vitro and in vivo biocompatibility and corrosion behaviour of a bioabsorbable magnesium alloy coated with octacalcium phosphate and hydroxyapatite. Acta Biomater..

[26] Noviana D., Paramitha D., Ulum M.F., Hermawan H. (2016). The effect of hydrogen gas evolution of magnesium implant on the postimplantation mortality of rats. J. Orthop. Transl..

[27] Song G. (2007). Control of biodegradation of biocompatable magnesium alloys. Corros. Sci..

[28] Shadanbaz S., Dias G.J. (2012). Calcium phosphate coatings on magnesium alloys for biomedical applications: A review. Acta Biomater..

